# Uncommon Origin, Common Presentation: A Rare Case of Mesenteric Carcinoid Tumor Mimicking Functional Gastrointestinal Disorders

**DOI:** 10.7759/cureus.93703

**Published:** 2025-10-02

**Authors:** Radhika Mathur, Rahul Borra, Salman Muddassir

**Affiliations:** 1 Internal Medicine, Hospital Corporation of America (HCA) | University of South Florida Morsani College of Medicine Graduate Medical Education (GME) Oak Hill Hospital, Brooksville, USA

**Keywords:** alpha-fetoprotein, carcinoid syndrome and rpf, chromogranin-a, ct-guided biopsy, enterochromaffin cells, metastasis, neuroendocrine cell tumor, sandostatin, synaptophysin, unexplained abdominal pain

## Abstract

Carcinoid tumors, a subtype of well-differentiated neuroendocrine neoplasms, are frequently misdiagnosed due to their indolent course and nonspecific gastrointestinal (GI) symptoms that mimic more common disorders. Here we report a rare case of a 59-year-old woman with hepatitis C and a history of intravenous drug use who presented with progressive abdominal pain, nausea, vomiting, diarrhea, and weight loss. Initial evaluations were inconclusive, but further imaging revealed a central mesenteric mass-an uncommon primary site-along with hepatic lesions, raising concern for metastatic disease. Elevated chromogranin A and biopsy confirmed a grade 1 neuroendocrine tumor. This case highlights the importance of considering carcinoid tumors in patients with persistent GI symptoms and emphasizes early recognition, particularly in atypical presentations, to guide timely and effective management.

## Introduction

Carcinoid tumors are well-differentiated neuroendocrine neoplasms (NETs) that arise from enterochromaffin cells and most commonly originate in the gastrointestinal tract-particularly the ileum, appendix, rectum, and stomach. While the bronchopulmonary system is the second most frequent site for NETs, primary mesenteric tumors are exceptionally rare, reported only in isolated cases [1,2], with most mesenteric involvement representing secondary disease. Population registries do not list mesenteric primaries separately, underscoring their rarity [3,4].

Despite their typically indolent behavior, carcinoid tumors possess malignant potential and frequently secrete bioactive substances, causing nonspecific symptoms such as abdominal pain, diarrhea, flushing, and weight loss [5]. These manifestations often mimic more common functional or inflammatory gastrointestinal disorders, which can lead to misdiagnosis and delayed recognition [6]. The incidence of carcinoid tumors has risen significantly over recent decades due to heightened clinical awareness and advances in diagnostic modalities. Serum chromogranin A remains a useful biochemical marker, while CT, MRI, and somatostatin receptor PET/CT provide valuable information for localization and staging [7,8]. Histopathological evaluation remains essential, with tumor grade and Ki-67 index serving as critical prognostic indicators [9]. Although slow-growing, carcinoid tumors frequently metastasize to the liver, significantly impacting prognosis and management strategies. Treatment options include surgical resection when feasible, somatostatin analogs for tumor stabilization and symptom control, and systemic therapies in advanced or progressive disease [10,11]. 

Of note, tumors in unusual sites like the mesentery pose a diagnostic challenge, as extraluminal lesions often lack early obstructive symptoms and can mimic desmoid tumors, lymphoma, or metastases on imaging [12]. Furthermore, in patients with comorbidities such as treated hepatitis C, overlapping hepatic-related complaints-including abdominal discomfort, fatigue, or abnormal liver imaging-can obscure the clinical picture and delay recognition of a neuroendocrine tumor. This overlap highlights the need for a high index of suspicion and a systematic approach to distinguishing hepatic pathology related to chronic viral infection from metastatic disease arising from mesenteric NETs. 

This case underscores the importance of considering carcinoid tumors in the differential diagnosis of persistent or unexplained gastrointestinal symptoms, particularly when extraluminal mesenteric lesions are identified. It also illustrates how concurrent conditions like hepatitis C may confound the diagnostic pathway [13], emphasizing the critical role of histopathology and comprehensive imaging in reaching a timely and accurate diagnosis.

## Case presentation

A 59-year-old woman with a medical history significant for intravenous drug use and successfully treated hepatitis C presented to the emergency department with a two-month history of progressively worsening, crampy, generalized abdominal pain. Her symptoms were accompanied by persistent nausea, intermittent vomiting, watery diarrhea, early satiety, and a 20-pound unintentional weight loss. She denied hematochezia, melena, fevers, chills, or jaundice. Her physical examination was notable for diffuse abdominal tenderness without rebound or guarding, mild distention, and trace pedal edema. Vital signs were within normal limits.

Given the vague and nonspecific nature of her symptoms, initial clinical impressions included chronic liver disease, functional bowel disorder, or occult infection. She had undergone a colonoscopy nine years earlier that was unremarkable, and there was no family history of gastrointestinal malignancy. Basic laboratory evaluation was non-revealing, with normal white blood cell count, liver enzymes, and lipase. However, given the persistence and severity of her symptoms, particularly the significant weight loss, cross-sectional imaging was pursued.

**Figure 1:**
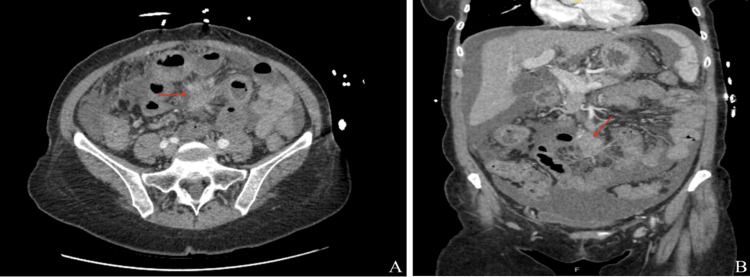
Axial (A) and sagittal (B) views of CT abdomen/pelvis demonstrating an ill-defined mesenteric soft tissue mass

Contrast-enhanced computed tomography (CT) of the abdomen and pelvis revealed an ill-defined central mesenteric soft tissue mass measuring 2.1 × 2.2 × 3.6 cm (Figures 1A, 1B), associated with radiating fibrotic strands and tethering of adjacent small bowel loops. The imaging appearance was suspicious for a desmoplastic reaction, raising concern for a carcinoid tumor. Other differential considerations included desmoid tumor, lymphoma, or metastatic disease. Additional findings included omental stranding, moderate ascites, cholelithiasis, common bile duct dilation up to 12 mm, and a nodular liver contour suggestive of underlying cirrhosis.

**Figure 2:**
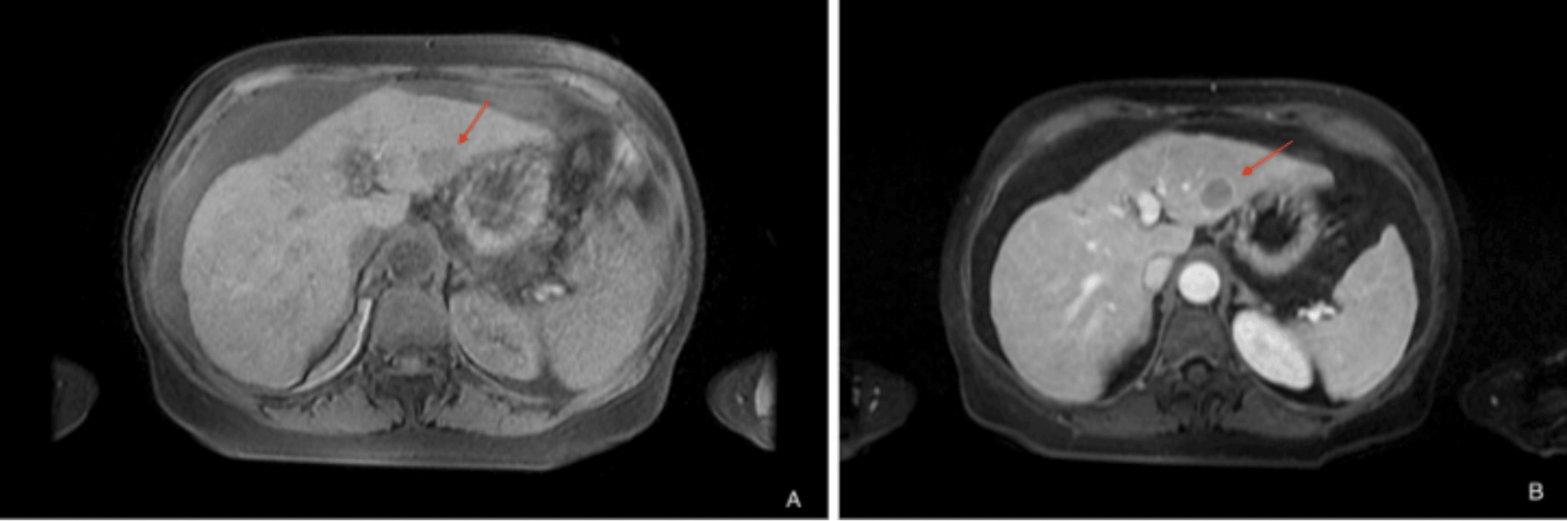
T1 fat saturated sequence showing ill-defined mass in the left hepatic lobe (A), followed by arterial enhancing mass in the left hepatic lobe measuring 4 mm without washout or capsule (B)

At this point, the diagnosis remained uncertain. The central location of the mass in the mesentery, rather than the bowel wall, further complicated the identification of a primary tumor site, making this an unusual presentation. Gastroenterology was consulted and recommended further evaluation with tumor markers, including alpha-fetoprotein (AFP) and chromogranin A, as well as magnetic resonance imaging (MRI) of the abdomen and magnetic resonance cholangiopancreatography (MRCP). MRI/MRCP confirmed the presence of the liver metastasis (Figure 2A) and revealed multiple arterially enhancing sub-centimeter hepatic lesions, the largest measuring 4 mm (Figure 2B).

AFP was within normal range (3.6 ng/mL), while chromogranin A was markedly elevated at 114.9 ng/mL, heightening suspicion for a neuroendocrine tumor. 

**Figure 3:**
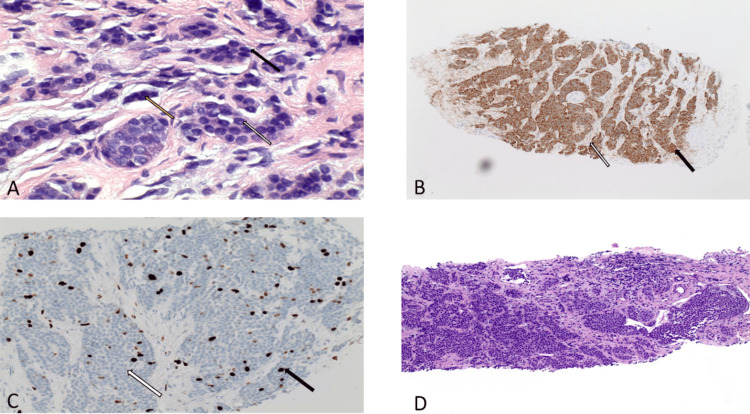
(A) Higher power demonstrating uniform cells with increased nuclear to cytoplasmic ratio (black arrow), smudged chromatin (yellow arrow) and inconspicuous nucleoli (white arrow); (B) synaptophysin immunohistochemical stain which is another marker of neuroendocrine differentiation (black arrow), fibrous septa (white arrow); (C) overview image of Ki- 67 staining where brown stain indicates actively dividing cells (black arrow), while blue staining (usually from hematoxylin) indicates cells that are not actively dividing (white arrow); (D) biopsy from the liver showing metastatic well-differentiated neuroendocrine tumor

CT-guided biopsy of the mesenteric mass revealed cords and nests of small, uniform cells with increased nuclear-to-cytoplasmic ratios (Figure 3A) and characteristic “salt-and-pepper” chromatin. Immunohistochemistry demonstrated positivity for neuroendocrine markers CD56 and synaptophysin (Figure 3B), with a low Ki-67 proliferation index (Figure 3C) consistent with a grade 1 well-differentiated neuroendocrine tumor. CDX2 staining suggested a lower gastrointestinal origin. Biopsy of a hepatic lesion confirmed metastatic disease with identical histopathology (Figure 3D). 

The patient was diagnosed with a metastatic grade 1 well-differentiated neuroendocrine tumor (carcinoid tumor) of probable lower GI origin, presenting with a primary mesenteric mass, a rare and often under-recognized site. Given the low Ki-67 index, indicative of a slow-growing, low-grade tumor with limited expected benefit from systemic chemotherapy, she was initiated on subcutaneous octreotide (Sandostatin) 100 mcg twice daily for symptom control and tumor stabilization. Palliative care consultation and close outpatient surgery and oncology follow-up were also recommended to optimize symptom management and guide long-term monitoring of her indolent disease.

## Discussion

Carcinoid tumors are slow-growing neuroendocrine neoplasms that frequently present with vague gastrointestinal complaints, often mimicking more common conditions and leading to diagnostic delays [3]. This case of a 59-year-old woman with a history of intravenous drug use, treated hepatitis C, and chronic, nonspecific gastrointestinal symptoms underscores the importance of maintaining a high index of suspicion for carcinoid tumors-particularly when imaging identifies a mesenteric mass with concurrent hepatic lesions. Extraluminal mesenteric tumors may not cause early obstructive symptoms, and their radiologic features can overlap with other pathologies such as desmoid tumors, lymphoma, or metastatic disease, complicating diagnosis [12]. In this context, underlying liver disease related to hepatitis C may obscure recognition of hepatic metastases, creating an additional diagnostic challenge. Nevertheless, liver metastases are common in carcinoid tumors and have a significant impact on both prognosis and management [8].

A review of the literature highlights both the rarity and complexity of this presentation. While carcinoid tumors most commonly arise in the small intestine, appendix, rectum, or stomach, primary mesenteric carcinoid tumors are exceptionally rare, reported only in isolated cases [1,2]. Chronic hepatitis C has been associated with gastric and small intestinal carcinoid tumors, suggesting that long-standing liver disease may indirectly influence tumor development or detection [13]. Although direct associations with intravenous drug use are limited, its link to immunocompromised states may increase susceptibility to malignancies, including NETs. Taken together, these factors underscore the diagnostic difficulty in patients with overlapping comorbidities and atypical tumor sites.

Diagnosis should follow a systematic, guideline-based approach. Biochemical evaluation, including serum chromogranin A and 24-hour urinary 5-hydroxyindoleacetic acid (5-HIAA), is recommended to support the suspicion of a neuroendocrine tumor and to monitor disease progression [4]. Cross-sectional imaging with contrast-enhanced CT or MRI of the abdomen and pelvis is essential for localizing the primary tumor and evaluating for mesenteric or hepatic involvement. Functional imaging, such as somatostatin receptor PET/CT, further refines staging and treatment planning. Histopathological confirmation through image-guided biopsy remains the gold standard, as demonstrated in this case by a CT-guided biopsy confirming a well-differentiated, grade 1 neuroendocrine tumor. The Ki-67 proliferative index provides key prognostic information and guides therapeutic decision-making; in this patient, a low Ki-67 index (<3%) reflected an indolent course with anticipated responsiveness to somatostatin analog therapy. 

Therapeutically, somatostatin analogs such as octreotide remain the cornerstone of management for symptom control and disease stabilization in metastatic carcinoid tumors [9]. Current National Comprehensive Cancer Network (NCCN) and European Neuroendocrine Tumor Society (ENETS) guidelines recommend somatostatin analogs as first-line therapy for unresectable or metastatic small intestinal and mesenteric NETs [14,15]. In cases of disease progression, additional treatment modalities, including peptide receptor radionuclide therapy (PRRT), everolimus, or liver-directed interventions, should be considered. 

This case highlights the critical need to consider carcinoid tumors in patients with persistent, unexplained gastrointestinal symptoms and suspicious imaging findings. Prognosis is strongly influenced by tumor grade, stage, and proliferative index. For mesenteric NETs specifically, survival outcomes are less favorable than for other gastrointestinal NETs: large series report five-year overall survival ranging from ~46-75% in patients with metastatic small intestinal NETs with mesenteric involvement, with significantly reduced survival when large mesenteric tumor deposits are present (65.5% vs. 92.6% without deposits), and a median survival of approximately 52 months [11].

## Conclusions

This case underscores the diagnostic challenge posed by neuroendocrine tumors, especially when presenting with nonspecific gastrointestinal symptoms and the exceptionally rare primary mesenteric location. A history of hepatitis C and intravenous drug use could have further complicated disease progression and recognition, reflecting how comorbidities may obscure the clinical picture. Early diagnosis through guideline-directed biochemical, radiologic, and histopathologic evaluation is crucial, as hepatic metastases are common and carry significant prognostic weight. Prompt initiation of somatostatin analog therapy, particularly in low-grade tumors, can stabilize disease and improve quality of life. Clinicians should therefore maintain a high index of suspicion for neuroendocrine neoplasms in patients with persistent, unexplained GI complaints, even when alternative explanations seem likely.
